# Supplementary material for: Mantle hydration and the role of water in the generation of large igneous provinces
Source: Nat Commun. 2017 Nov 28;8:1824. doi: 10.1038/s41467-017-01940-3 (PMC5704025; doi:10.1038/s41467-017-01940-3)
Supplement: Supplementary file 1 — Supplementary Information [file 41467_2017_1940_MOESM1_ESM.pdf]

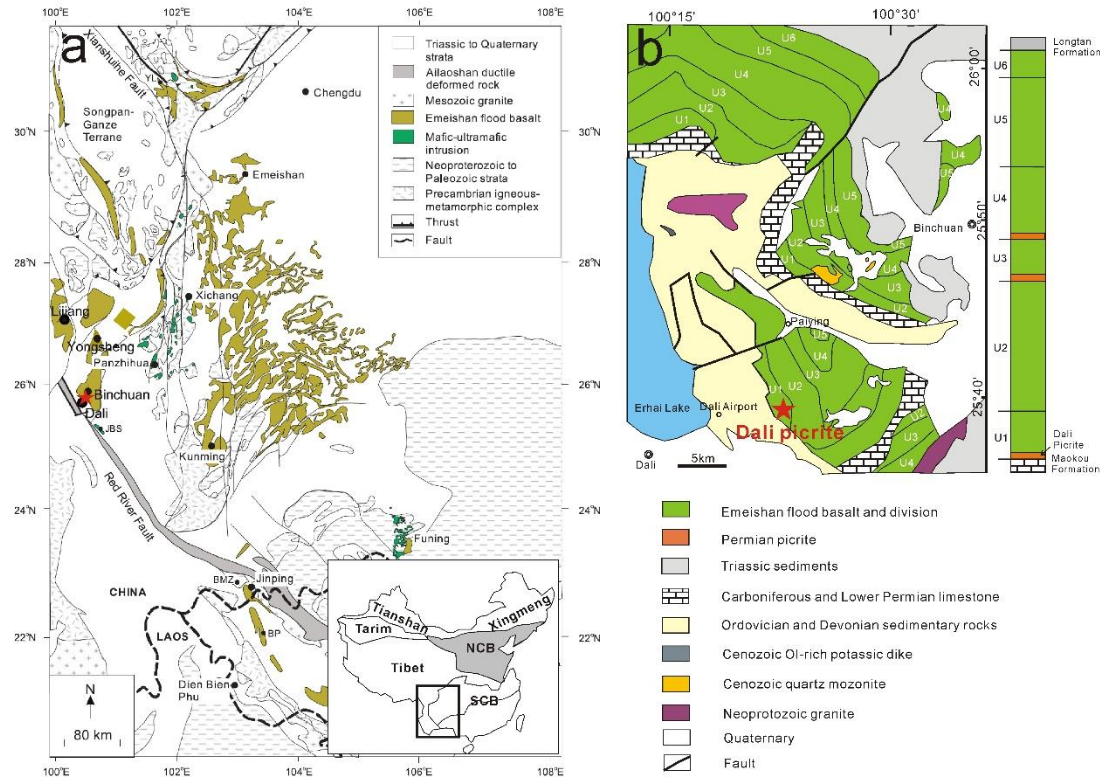

Supplementary Figure 1. a, Map showing the distribution of volcanic and intrusive rocks in the Emeishan large igneous province (ELIP), SW China. **b,** Location of the sampling site of Dali picrite east of Lake Erhai and in a stratigraphic column of ELIP. The maps in **a** and **b** are after ref. [1] and ref. [2].

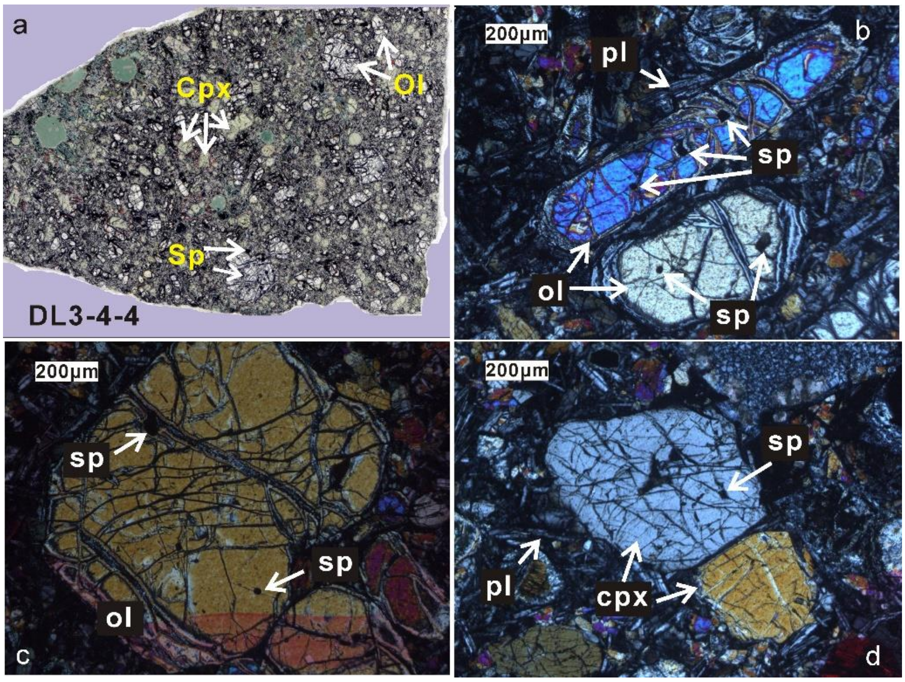

Supplementary Figure 2. a, Scanned thin section of Dali picrite sample DL3-4. **b-d**, Photomicrographs of olivine (ol), clinopyroxene (cpx) and chrome spinel (sp) phenocrysts in cross-polarised light. Note that many olivine phenocrysts contain spinel inclusions. Pl is plagioclase microlite. Same sample as in **a**.

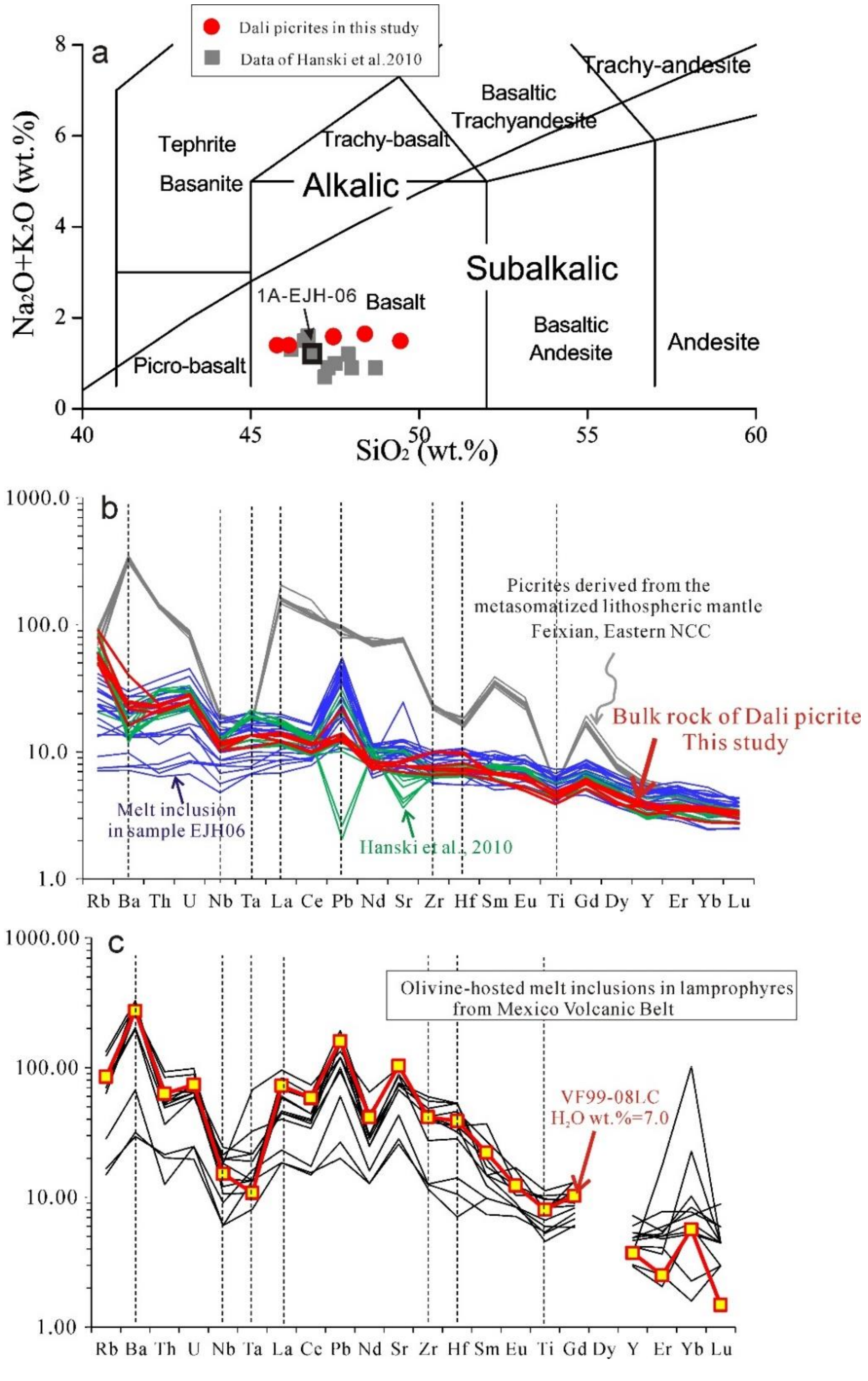

Supplementary Figure 3. Major and trace element characteristics of Dali picrites. **a,** TAS diagram. Data from ref. [3] are shown for comparison. Sample 1A-EJH-06 was used for analysis of melt inclusions in olivine phenocrysts. **b,** Primitive mantle-normalised trace element patterns for Dali picrites. The patterns of Feixian picrites in North China Craton, which were derived from metasomatised lithospheric mantle and contained about >3 wt.% water[4,5], are shown for comparison. Primitive mantle values are from ref.[6]. **c**, Primitive mantle-normalised trace element patterns of the melt inclusions hosted in olivine phenocrysts from lamprophyres in the Mexico Volcanic Belt. VF00-98LC is the code for a melt inclusion with a water content of 7.0 wt.%.

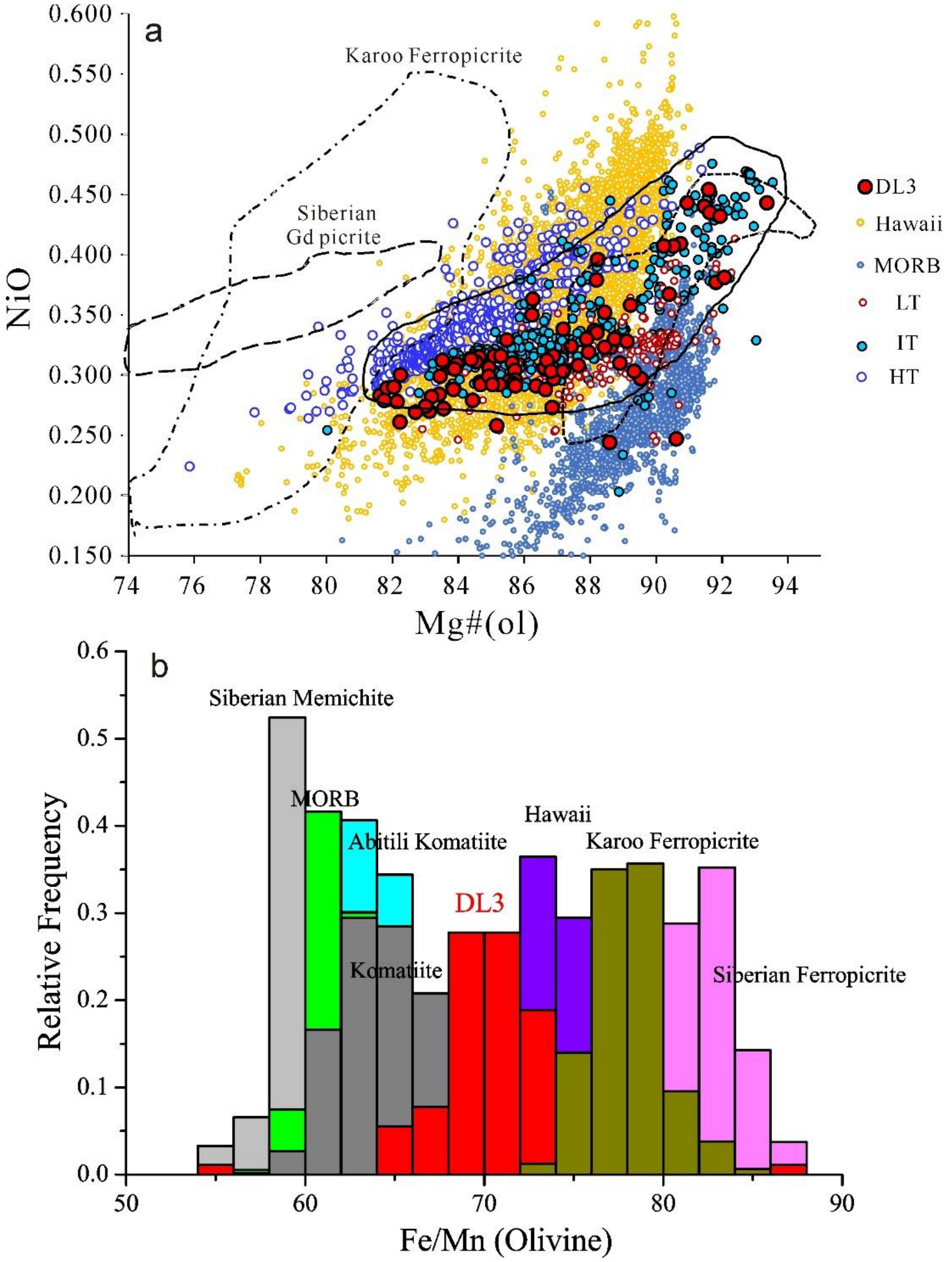

Supplementary Figure 4. NiO and Fe/Mn ratios by weight in olivine phenocrysts from Dali picrites compared with olivine data from other magmas. a, Plot of NiO versus Mg# of olivine. Hawaii (Koolau and Loihi seamount), MORB and komatiite (Gorgona, Alexo, Munro, and Belingwe, shown by the thin dashed line region) data are from ref.[7]. LT, HT and IT are for low-Ti, high-Ti and intermediate-Ti picrites from the Emeishan LIP, respectively, with data taken from ref.[1]. The black solid line marks the field for Dali picrites based on data from ref. [8]. Data for Karoo ferropicrite and Siberian Gudchikhinsky (Gd) picrite (the region shown by dot-dashed line and thick dashed line) are from ref. [9,10], respectively. **b,** Fe/Mn ratios in olivine of primitive magmas from different settings. Data sources the same as in **a**.

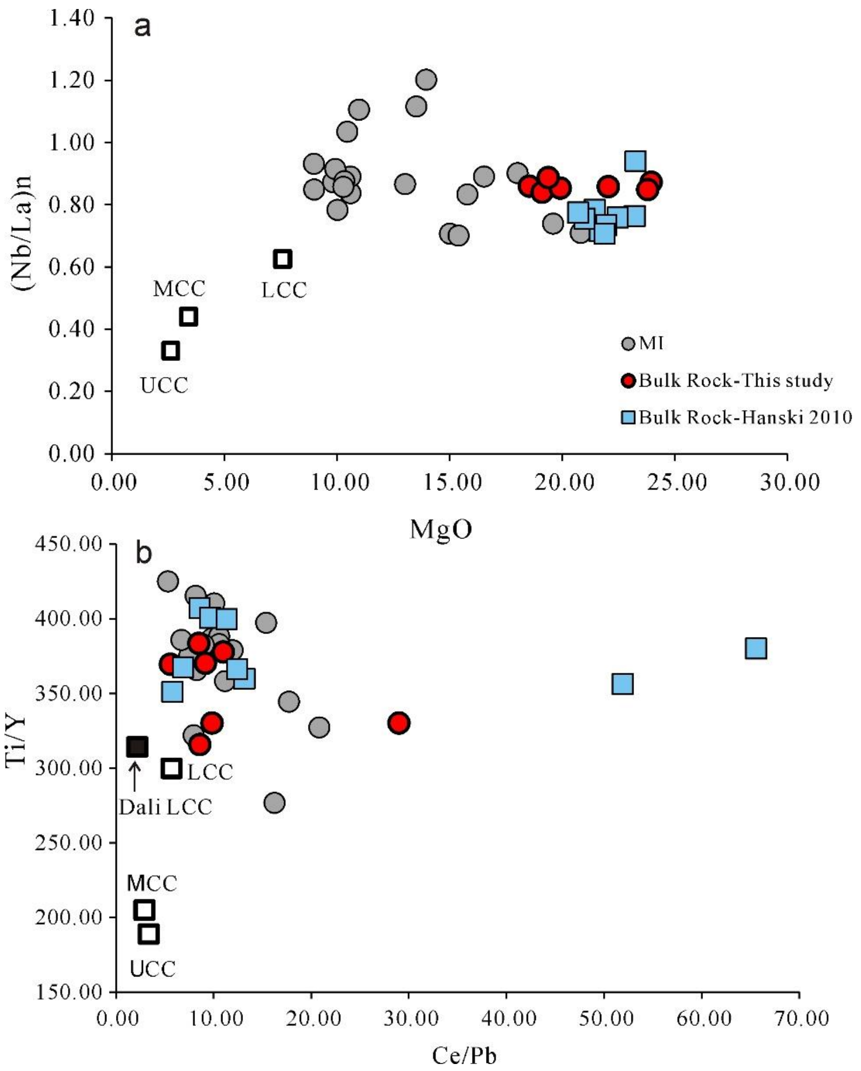

Supplementary Figure 5. a, MgO vs. (Nb/La)n plots for Dali picrites. b, Ti/Y vs. Ce/Pb plots for Dali picrites. MI, melt inclusions in olivine phenocrysts. The open squares represent the compositions of upper continental crust (UCC), middle continental crust (MCC) and lower continental crust (LCC) after ref. [11]. The data of the lower continental crust xenoliths in Dali (Dali LCC) is from ref. [12].

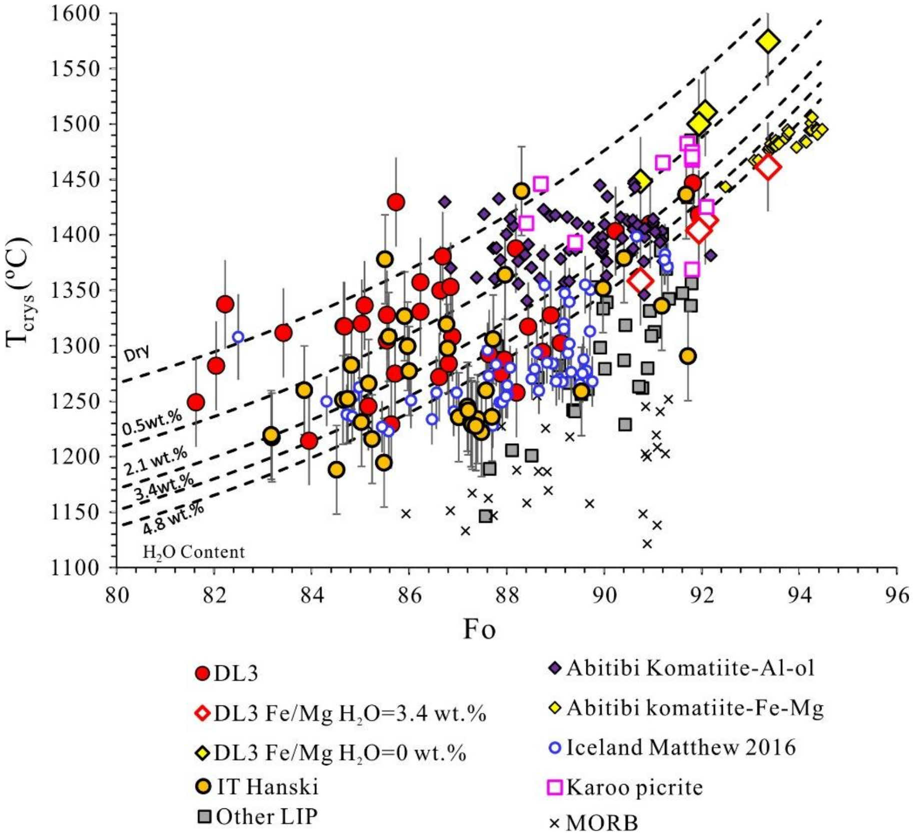

Supplementary Figure 6. Crystallization temperature versus olivine composition diagram for Dali picrites and other primitive magmas. The temperatures were calculated using the thermometer based on Al partitioning between olivine and spinel[13] and Fe-Mg equilibrium between olivine and melt[14]. The red and orange filled circles are for the Al thermometer and diamonds for the Fe-Mg thermometer, respectively, with the data taken from this work and Hanski et al. (2010). The Karoo picrite data are from ref. [15], the Abitibi komatiite data from ref. [16] and other LIP and MORB data from ref. [13]. The dashed lines represent the temperature–composition relationship of olivine in the primary melt of Dali picrite (DL3-1 in this study) at 1.3 GPa with initial water contents of 0, 0.5, 2.1, 3.4 and 4.8 wt.%, calculated using the models of ref. [17,18].

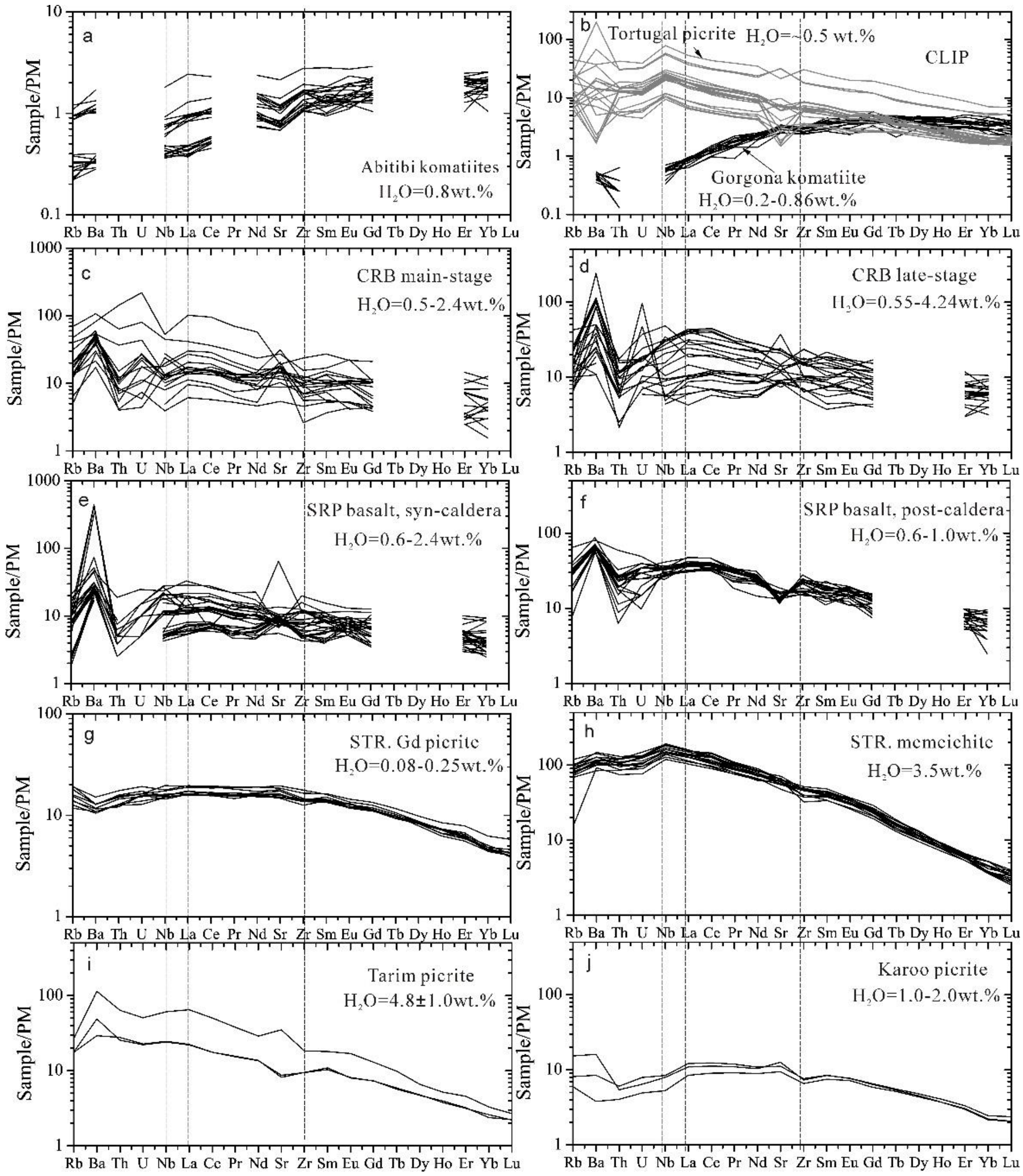

Supplementary Figure 7. Primitive mantle-normalised trace element patterns for hydrous primary magmas from continental LIPs. CLIP, Caribbean LIP; CFB, Columbia River basalts; SRP, Snake River Plain basalts; STR, Siberian Trap. Data sources: Abitibi komatiite from ref.[16] , Gorgona komatiites from ref.[19], Tortugal picrite from ref.[20]; Columbia River basalts (CFB) from ref.[21], Gd Formation picrites and meimechite in Siberian Trap (STR) from ref.[22,23], Tarim picrite from ref.[24], and Karoo picrite from ref.[9,25]. The water content of Tarim basalt is from ref.[26].

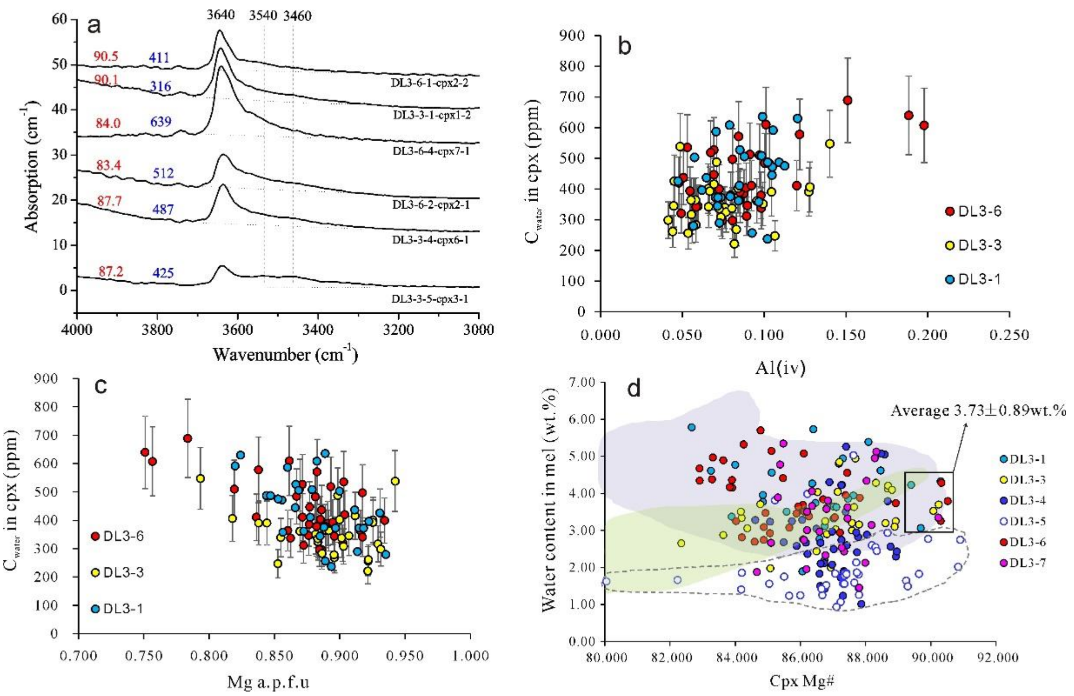

Supplementary Figure 8. OH bands and major elemental characteristics of cpx phenocrysts in Dali picrite. **a**, Typical FTIR bands of OH in cpx phenocrysts from Dali picrites. The dotted lines show the spline baseline for the water content calculations. The red and blue numbers on the spectra are Mg# and H_2O (ppm) of cpx, respectively. **b,c,** Comparison of water contents and cation abundances in cpx phenocrysts from Dali picrites. The Al(iv) and Mg atom numbers were calculated on the basis of 6 oxygens in the pyroxene formula. Water contents of cpx were measured by unpolarised FTIR with an uncertainty of less than 30%. **d**, Calculated water content in the melt in equilibrium with cpx phenocrysts from Dali picrites. The light blue and green fields are the calculated water contents in melts for basalts from the Iraz ú and Arenal volcanos, Costa Rica. The water content and major element data for cpx are from ref.[27], and the water partition coefficients were calculated according to the calibration by ref.[28]. The field defined by the dashed line marks the cpx population with a lower water content than that in DL3-3 and DL3-1. These low water contents were most likely caused by diffusional loss after cpx crystallization, as demonstrated by the diffusion profile in Supplementary Figure 9. The rectangle outlines the cpx phenocrysts that were used for calculating the water content in the “primary” magma.

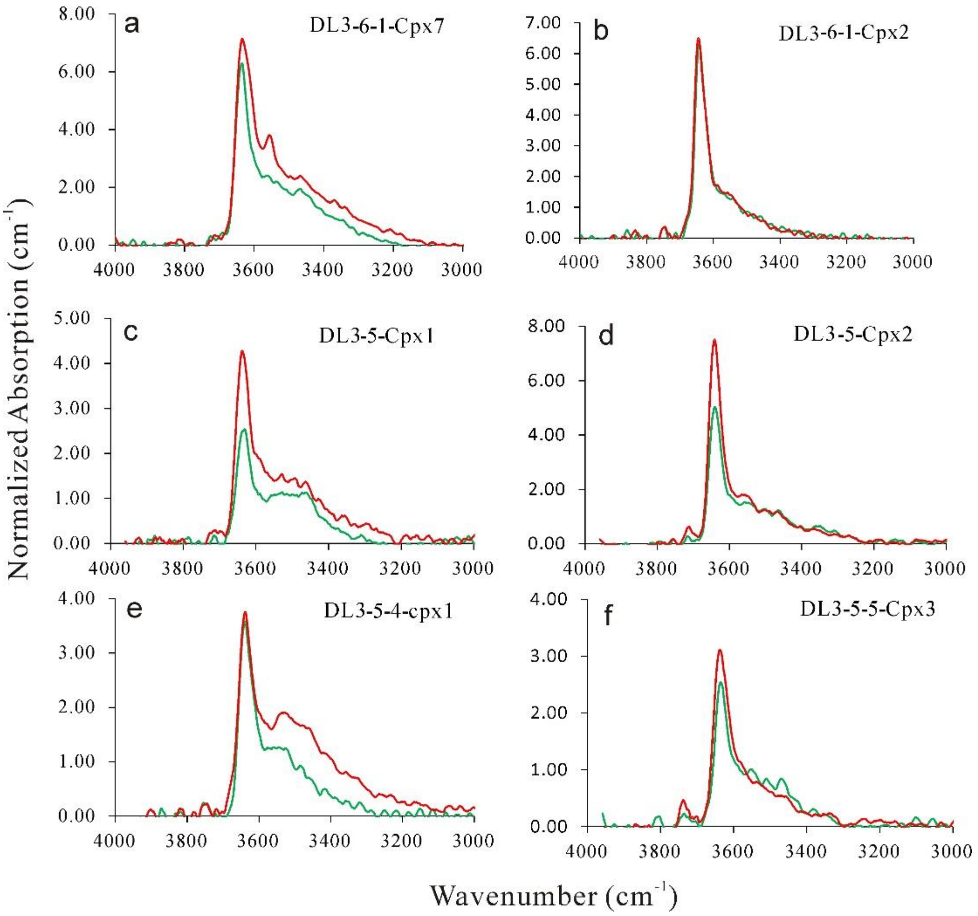

Supplementary Figure 9. Results of FTIR profile analyses for cpx phenocrysts from Dali picrites. a and **b** are for sample DL3-6 and **c-f** are for sample DL3-5. The red and green spectra are for the core and rim of cpx, respectively. All spectra have been corrected for baseline, as indicated in Supplementary Figure 8a.

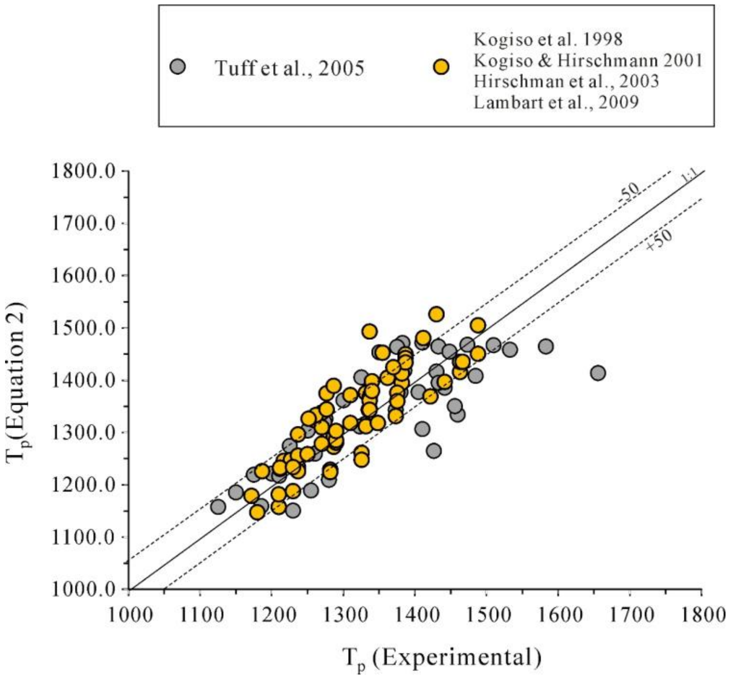

Supplementary Figure 10. Test of T_P estimation for pyroxenite sources by the peridotite-based model of ref.[17]**.** The experimental T_P was calculated with the experimental pressure and the mantle adiabatic gradient (13.3 ℃/GPa; ref.[14]). **a**, T_P (Equation 2) was calculated based on Equation (3) and Equation (2) (Equation 13 and Equation 17 in ref.[17]). See Methods for the calculation detail and Supplementary Table 4 for the data.

Supplementary references:

1. Kamenetsky, V. S., Chung, S. L., Kamenetsky, M. B. & Kuzmin, D. V. Picrites from the emeishan large Igneous Province, SW China: A compositional continuum in primitive magmas and their respective mantle sources. *J. Petrol.* **53,** 2095–2113 (2012).
2. Tang, Q., Li, C., Zhang, M. & Lin, Y. U–Pb age and Hf isotopes of zircon from basaltic andesite and geochemical fingerprinting of the associated picrites in the Emeishan large igneous province, SW China. *Mineral. Petrol.* **109,** 103–114 (2015).
3. Hanski, E., Kamenetsky, V. S., Luo, Z. Y., Xu, Y. G. & Kuzmin, D. V. Primitive magmas in the Emeishan Large Igneous Province, southwestern China and northern Vietnam. *Lithos* **119,** 75–90 (2010).
4. Xia, Q. K. *et al.* High water content in Mesozoic primitive basalts of the North China Craton and implications on the destruction of cratonic mantle lithosphere. *Earth Planet. Sci. Lett.* **361,** 85–97 (2013).
5. Gao, S. *et al.* Recycling deep cratonic lithosphere and generation of intraplate magmatism in the North China Craton. *Earth Planet. Sci. Lett.* **270,** 41–53 (2008).
6. McDonough, W. F. & Sun, S.-s. The composition of the Earth. *Chem. Geol.* **120,** 223–253 (1995).
7. Sobolev, A. V *et al.* The amount of recycled crust in sources of mantle-derived melts. *Science* **316,** 412–417 (2007).
8. Ren, Z. *et al.* Primary magmas and mantle sources of Emeishan basalts constrained from major

element , trace element and Pb isotope compositions of olivine-hosted melt inclusions. *Geochim. Cosmochim. Acta* **208,** 63–85 (2017).

9. Heinonen, J. S. & Luttinen, A. V. Mineral chemical evidence for extremely magnesian subalkaline melts from the Antarctic extension of the Karoo large igneous province. *Mineral. Petrol.* **99,** 201–217 (2010).
10. Sobolev, A. V., Krivolutskaya, N. A. & Kuzmin, D. V. Petrology of the parental melts and mantle sources of Siberian trap magmatism. *Petrology* **17,** 253–286 (2009).
11. Rudnick, R. L. & Gao, S. *Composition of the Continental Crust*. *Treatise on Geochemistry: Second Edition* **4,** (Elsevier Ltd., 2013).
12. Wang, J., Li, J.-P., Wang, J.-H. & Ma, Z.-H. Geological implications for the mafic enclaves of deep deriveation from cenozoic shoshonitic rocks in Jianchuan-Dali area, Western Yunnan. *Acta Mineral. Sin.* **22,** 113–125 (2002).
13. Coogan, L. A., Saunders, A. D. & Wilson, R. N. Aluminum-in-olivine thermometry of primitive basalts: Evidence of an anomalously hot mantle source for large igneous provinces. *Chem. Geol.* **368,** 1–10 (2014).
14. Putirka, K. D., Perfit, M., Ryerson, F. J. & Jackson, M. G. Ambient and excess mantle temperatures, olivine thermometry, and active vs. passive upwelling. *Chem. Geol.* **241,** 177–206 (2007).
15. Heinonen, J. S., Jennings, E. S. & Riley, T. R. Crystallisation temperatures of the most Mg-rich magmas of the Karoo LIP on the basis of Al-in-olivine thermometry. *Chem. Geol.* **411,** 26–35 (2015).
16. Sobolev, A. V *et al.* Komatiites reveal a hydrous Archaean deep-mantle reservoir. *Nature* **531,** 628–632 (2016).
17. Herzberg, C. T. & Asimow, P. D. PRIMELT3 MEGA.XLSM software for primarymagma calculation: Peridotite primarymagma MgO contents fromthe liquidus to the solidus. *Geochem., Geophys. Geosys.* **16,** 563–578 (2015).
18. Falloon, T. J. & Danyushevsky, L. V. Melting of Refractory Mantle at 1.5, 2 and 2.5 GPa under Anhydrous and H2O-undersaturated Conditions: Implications for the Petrogenesis of High-Ca Boninites and the Influence of Subduction Components on Mantle Melting. *J. Petrol.* **41,** 257–283 (2000).
19. Gurenko, A. A., Kamenetsky, V. S. & Kerr, A. C. Oxygen isotopes and volatile contents of the Gorgona komatiites, Colombia: A confirmation of the deep mantle origin of H2O. *Earth Planet. Sci. Lett.* **454,** 154–165 (2016).
20. Trela, J. *et al.* The hottest lavas of the Phanerozoic and the survival of deep Archaean reservoirs. *Nat. Geosci.* (2017). doi:10.1038/ngeo2954
21. Cabato, J. A., Stefano, C. J. & Mukasa, S. B. Volatile concentrations in olivine-hosted melt inclusions from the Columbia River flood basalts and associated lavas of the Oregon Plateau: Implications for magma genesis. *Chem. Geol.* **392,** 59–73 (2015).
22. Sobolev, A. V., Sobolev, S. V., Kuzmin, D. V., Malitch, K. N. & Petrunin, A. G. Siberian meimechites: origin and relation to flood basalts and kimberlites. *Russ. Geol. Geophys.* **50,** 999–1033 (2009).
23. Sobolev, A. V., Krivolutskaya, N. A. & Kuzmin, D. V. Petrology of the parental melts and mantle sources of Siberian trap magmatism. *Petrology* **17,** 253–286 (2009).
24. Tian, W. *et al.* The Tarim picrite-basalt-rhyolite suite, a Permian flood basalt from Northwest

China with contrasting rhyolites produced by fractional crystallization and anatexis. *Contrib. Mineral. Petrol.* **160,** 407–425 (2010).

25. Heinonen, J. S. & Luttinen, A. V. Jurassic dikes of Vestfjella, western Dronning Maud Land, Antarctica: Geochemical tracing of ferropicrite sources. *Lithos* **105,** 347–364 (2008).
26. Xia, Q. *et al.* High water content in primitive continental flood basalts. *Sci. Rep.* **6**, doi:10.1038/srep25416 (2016).
27. Wade, J. A. *et al.* Prediction of magmatic water contents via measurement of H_2O in clinopyroxene phenocrysts. *Geology* **36,** 799–802 (2008).
28. O'Leary, J. A., Gaetani, G. A. & Hauri, E. H. The effect of tetrahedral Al3+ on the partitioning of water between clinopyroxene and silicate melt. *Earth Planet. Sci. Lett.* **297,** 111–120 (2010).
