# Supplementary material for: Mantle hydration and the role of water in the generation of large igneous provinces
Source: Nat Commun. 2017 Nov 28;8:1824. doi: 10.1038/s41467-017-01940-3 (PMC5704025; doi:10.1038/s41467-017-01940-3)
Supplement: Supplementary file 3 — Description of Additional Supplementary Files [file 41467_2017_1940_MOESM3_ESM.pdf]

Description of Additional Supplementary Files

File Name: Supplementary Data 1
Description: Compositions of Cr-spinel phenocrysts and inclusions in olivine and their host olivine from Emeishan LIP picrites.

File Name: Supplementary Data 2
Description: The major and trace element concentration of melt inclusions in olivine phenocrysts in Dali picrite (1A-EJH-06).

File Name: Supplementary Data 3
Description: The water content.

File Name: Supplementary Data 4
Description: The major element concentration of the starting materials in the partial melting experiments on pyroxenite.
